# Interaction of Blenoxane and Congeners Bleomycins A2 and B2 with Human Plasma Proteins Using Circular Dichroism Spectroscopy

**DOI:** 10.3390/ijms241713598

**Published:** 2023-09-02

**Authors:** Edoardo Longo, Giuliano Siligardi, Rohanah Hussain

**Affiliations:** B23 SRCD Beamline, Diamond Light Source Ltd., Harwell Science and Innovation Campus, Didcot, Oxfordshire OX11 0DE, UK; edoardo.longo@unibz.it (E.L.); giuliano.siligardi@diamond.ac.uk (G.S.)

**Keywords:** bleomycin, pharmacokinetic, protein complex stability, binding affinity, plasma proteins, acid glycoprotein, serum albumin

## Abstract

Bleomycin is a glycopeptide congeners’ family of antitumor antibiotics employed for the treatment of several types of tumors such as squamous cell carcinomas and malignant lymphomas. The general chemical structure is constituted by three main portions: (i) a metal binding domain that is recognized to be responsible for the DNA cleavage activity; (ii) a DNA binding domain via the 1-4’ bithiazole moiety; and (iii) a carbohydrate domain thought to be responsible for the accumulation of bleomycin in some cancer cells. To date, a limited number of protein interactions with bleomycin have been studied, but the plasma binding has not yet been determined. Here, we explore this aspect of the protein binding capacity of bleomycin to the two most abundant plasma proteins, human serum albumin (HSA) and α1-acid glycoprotein (AGP), which are known to bind and to be carriers of many drug molecules using spectroscopic techniques, such as circular dichroism, UV-vis absorbance, and fluorescence. The results showed that bleomycin binds to plasma proteins with an order-of-magnitude higher affinity for AGP than HSA. This is particularly important as AGP is an acute phase protein and is overexpressed in cancer patients. This should be taken into consideration as it could affect the therapeutic effect of the bleomycin dosage.

## 1. Introduction

**Figure 1:**
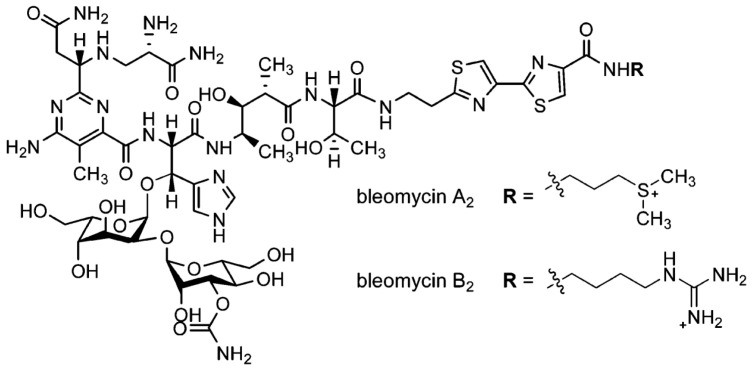
General structure of Bleomycin and C-terminal R substitution for Blm-A2 and Blm-B2 congeners.

The antibiotic family of congeners known as bleomycins (commercialized as Blenoxane^®^) has gained interest in the last 60 years due to its antitumor properties as a single- and double-strand DNA scissor [1,2]. Since its discovery in 1966 (by Umezawa and co-workers) [3], its chemistry, structure, metals, and DNA binding properties have been investigated using techniques such as NMR, X-ray crystallography, absorbance, fluorescence, circular dichroism (CD), and magnetic circular dichroism (MCD). A large part of the chemistry of bleomycins, particularly with respect to DNA interactions, has been understood, and their achievements have been reviewed [2,4,5]. In addition to the in-cell chemistry (the ability to cut the DNA in the presence of oxygen and iron cofactors), the interaction of bleomycin with membrane proteins has been investigated to elucidate the transport mechanism across the cell membrane [6,7,8,9,10,11,12,13]. In these terms, the information on the availability and transport of the drug to its target may need to be investigated more thoroughly. For example, information on the plasma protein interactions of bleomycin is scarce [7,14,15,16]. CD is useful to study protein–ligand interactions [17,18,19,20] as it is sensitive to the protein secondary structure that can be affected by ligand binding interactions. In the far-UV range (180–260 nm), CD can efficiently measure the secondary structure content of proteins as each type of folding displays characteristic signatures that can be estimated using several algorithms, such as CONTIN, CDSSTR, SELCON3 [21], and BeStSel [22]. In the near-UV range (260–340 nm), aromatic side chains and disulfide bonds are the chromophores that generate the CD, from which the phenylalanine, tyrosine, and tryptophan amino acid residues can be used as native probes to sense ligands within a short range of up to 6 Å and can also be used effectively to determine, qualitatively and quantitatively, ligand binding interactions by conducting CD titrations [23,24]. This study is to show how bleomycins interact with the two most abundant plasma proteins, human serum albumin (HSA) and α1-acid glycoprotein (AGP). HSA is responsible for the transport of drugs in the blood stream and the regulation of pharmacokinetic [22]. AGP, on the other hand, is an acute reactive plasma protein whose concentration can be increased tenfold or more in cancer patients [25,26], and binding to AGP could reduce the effectiveness of the cancer drug bleomycin. In this study, we applied several techniques based on CD developed at the B23 beamline (Diamond Light Source Ltd., Oxfordshire, UK) to investigate the human plasma binding proteins HSA and AGP to Blenoxane and the two main bleomycin congeners A2 and B2 (Figure 1) [9].

These analogues are endowed with almost identical structures but differ at the C-terminus end, where Blm-A2 has a dimethyl sulphonium group while Blm-B2 has a guanidinium instead [3]. We have monitored the light and thermal stability of AGP and HSA with and without the presence of commercial bleomycin, as well as the two isolated congeners and their binding affinity, with the results indicating that these congeners bind with different dissociation constants to the proteins.

## 2. Results

The binding interactions of bleomycins with HSA and AGP, respectively, were determined as follows:

(i) By assessing qualitatively spectral differences between the observed spectra of the mixtures of proteins with ligands and the calculated spectra from the sum of the spectra of the individual components in the same molar ratio of the mixtures;
(ii) By determining quantitatively the dissociation constant Kd from CD titrations data;
(iii) By determining the thermodynamic parameters Tm, ΔH, and ΔS and comparing the proteins alone and the protein–ligand mixtures as a function of temperature [17,18,19,20];
(iv) By using a photo denaturation assay to determine the rate of denaturation as a function of UV irradiation. This was carried out by exploiting the high photo flux of B23 beamline for SRCD via scanning 30 repeated consecutive spectra in the far-UV region (180–260nm) of Blenoxane, and bleomycin A2 and B2 with plasma proteins HSA and AGP, which is unattainable using bespoke benchtop CD instruments [24].

**Figure 2:**
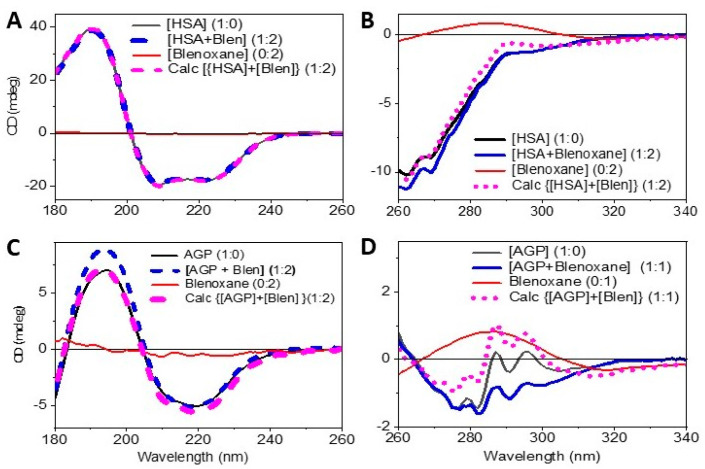
CD spectra of [HSA + Blenoxane] at molar ratio (1:2), HSA (1:0), and [Blenoxane] (0:2), and the sum of HSA spectrum with that of Blenoxane {Calc [HSA (1:0] + [Blen (0:2)]} in the far-UV region (**A**) and near-UV region (**B**). CD spectra of [AGP + Blenoxane] (1:n), [AGP] (1:0), [Blenoxane] (0:n), and the sum of AGP spectrum with that of Blenoxane {Calc [AGP (1:0] + [Blen (0:n)]} in the far-UV region (molar ratio 1:2) (**C**) and near-UV region (molar ratio 1:1) (**D**).

**Figure 3:**
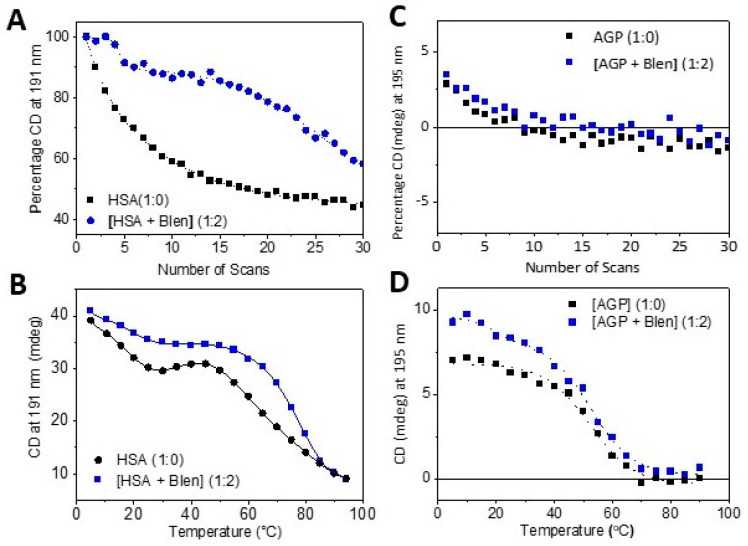
[HSA] (1:0) (black) and [HSA + Blenoxane] (1:2) (blue): (**A**) UV denaturation plot of percentage of SRCD at 191 nm versus number of 30 repeated consecutive scans in the far–UV region (180–260 nm), and (**B**) thermal denaturation plot of CD changes at 191 nm versus temperature. [AGP] (1:0) (black) and [AGP + Blenoxane] (1:2) (blue): (**C**) UV denaturation plot of percentage of SRCD at 195 nm versus number of 30 repeated consecutive scans in the far–UV region (180–260nm), and (**D**) thermal denaturation plot of CD changes at 195 nm versus temperature.

The observed SRCD spectrum of [HSA + Blenoxane (1:2)] compared to that calculated from the sum of the spectrum of HSA alone with that of Blenoxane alone was perfectly superimposable (Figure 2A), indicating that from the point of view of the secondary structure, there were no detectable conformational changes for HSA in the presence of Blenoxane. The same measurements carried out in the near-UV region, which is a characteristic of the local environment of amino acid aromatic residues Phe (around 260 nm), Tyr (around 280 nm), and Trp (around 290 nm) and disulfide dihedral angle (>300 nm) or any other chromophore that absorbs in the near-UV region [27,28], showed visible changes in the CD spectra. The UV denaturation (Figure 3A) and thermal denaturation (Figure 3B) showed significant differences in both the rate and the mode of denaturation for HSA in the presence of bleomycin.

Unlike HSA, a significant secondary structure conformational change was observed for [AGP + Blenoxane] (1:2)] compared to the calculated mixture at the same molar ratio in the far-UV (Figure 2C) and near-UV (Figure 2D) regions. On the other hand, negligible changes were observed for the UV photo denaturation assay between the AGP and AGP with Blenoxane (Figure 3C) and a marginal one for the thermal denaturation (Figure 3D), revealing how a single type of experiment used to diagnose protein–ligand binding interactions can be misleading. The CD of bleomycin with HSA in the far-UV region did not show any spectral change upon titration with bleomycin at an increased molar ratio, indicating either no binding interactions or binding interactions that did not affect the secondary structure. To determine whether binding interactions were occurring, titration was conducted in the near-UV region. Small but significant changes were observed that were qualitatively diagnostic of the binding interactions, but as the magnitude of the changes were rather small, qualitatively, it was not possible to determine the Kd accurately. The Kd was determined instead by fluorescence as the spectral changes upon titration were big enough for an accurate calculation. This is an example of where two label-free spectroscopic techniques, in this case CD and fluorescence, are necessary to characterize, unambiguously, the binding interactions and accurately determine the Kd of protein–ligand interactions in solution. Thermal stability is another method used to qualitatively determine whether there are ligand binding interactions to proteins and nucleic acids. Isothermal calorimetry (ITC) is another method used to determine qualitatively and quantitatively these interactions, though one must be aware that if the ligand displays bound water molecules in the binding site, the heat generated and absorbed might cancel out, meaning that the interaction will not be revealed. For the immobilized system, such as surface plasmon resonance (SPR) and fluorescence labelled systems, the risk of positive and negative false results must be taken into consideration. In this respect, CD spectroscopy does not have this limitation, and a bonus one would be able to see whether backbone conformational changes occur and what type of conformation is induced or not by the ligand interaction in the far-UV region or changes in the local environment of amino acid aromatic side chains and/or changes in the dihedral angles of the disulfide bonds [28,29].

**Figure 4:**
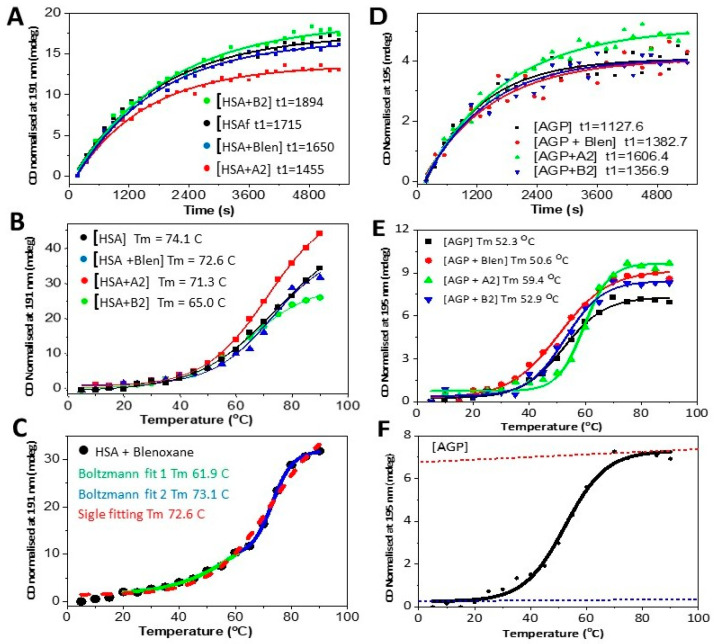
HSAff with and without bleomycins: (**A**) UV denaturation, ellipticity change. Fitting equation y = A1exp (−x/t1) + y0. Adj R-Square: 0.9909, 0.98993, 0.99016, and 0.99513, respectively. Data point calculated as change in ellipticity (in analogy with the Boltzmann plot hereafter). (**B**) Thermal denaturations. Boltzmann fitting of Tm experiment decay plot at 191 nm for HSA with and without Blenoxane, A2 and B2. (**C**) HSA with Blenoxane. The related thermodynamic parameters are reported in Table 1 for the various fittings. AGP with and without bleomycins: (**D**) UV denaturation, ellipticity changes. Fitting equation y = A1exp (−x/t1) + y0. Adj R-Square: 0.9909, 0.98993, 0.99016, and 0.99513, respectively. (**E**) Thermal denaturations. Boltzmann fitting of Tm experiment decay plot at 195 nm. (**F**) Example of linear extrapolation at T >> Tm and T << Tm.

In Figure 4, the UV denaturation and thermal denaturation plots for HSA and AGP with and without Blenoxane, and the two congeners A2 and B2, are shown, from which the thermodynamic parameters and UV denaturation rates are reported in Table 1 and Table 2, respectively.

In Table 1, the Tm is evaluated by linear regression, applying a single Boltzmann model. The Tm of AGP appeared to be affected by the presence of A2, showing also distinct differences in both enthalpy and entropy compared to the other congener B2 and Blenoxane.

For HSA, no big differences were displayed, except in the lower T_m_ for the [HSA + B2] complex and for [HSA + Blenoxane] (Figure 4C), for which a separate fitting of the initial and final part of the curve (Figure 4C) presented fitted parameters (Tm) much closer to the two values obtained with the two separated congeners. For AGP, the differences displayed were greater (Figure 4D,E) both in terms of fitting and estimated parameter values. For the UV denaturations, [AGP + A2] showed a t_1/2_ value (time scale of the denaturation process) much longer than that for [AGP + B2]. The same results were obtained from the thermal denaturation experiments, where the difference in the Tm was about 8–9 °C. From the fitting curves, the half-lives of the UV photo denaturations are calculated and reported in Table 2.

With AGP, the changes throughout the measurements appeared to indicate a certain uniformity. Nonetheless, the t_1/2_ for [AGP + A2] was higher than [AGP + B2], [AGP + Blenoxane], and AGP alone.

**Figure 5:**
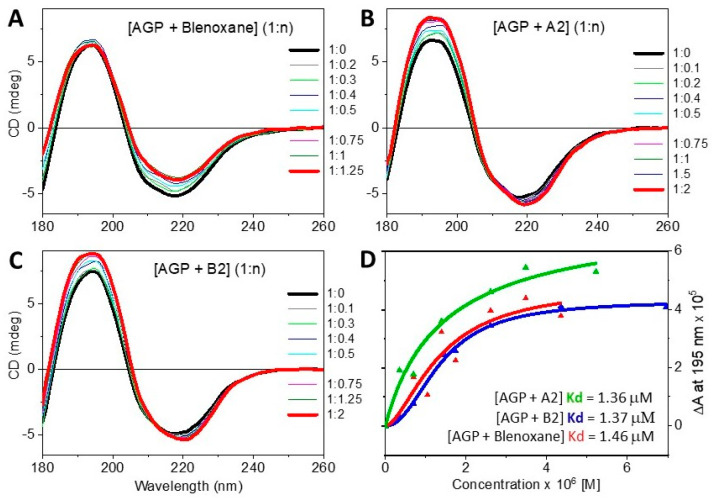
CD spectra of AGP titration: (**A**) [AGP + Blenoxane] (1: n); (**B**) [AGP + A2] (1: n); (**C**) [AGP + B2] (1: n). (**D**) CD titrations plots of CD reported in ΔA at 195 nm versus ligand concentration: [AGP + Blenoxane] (red), [AGP + A2] (green) and [AGP + B2] (blue).

CD titrations were carried out in the far-UV region for AGP with Blenoxane and A2 and B2 congeners to determine the dissociation constant Kd, analyzing the CD data with the Hill1 equation of OriginPro. The Kd appeared to be very similar among the three peptide-based ligands in the 1–2 μM range (Table 3), indicative of tight binding affinities: Kd of 1.36 (±0.82) ·10^−6^ M with Blenoxane, 1.46 (±1.33) ·10^−6^ M with A2, and 1.37 (±0.06) ·10^−6^ M with B2 (Figure 5).

In the near-UV regions, small CD spectral changes upon the titration of Blenoxane, A2, and B2 to AGP were observed (Figure 2D), which were consistent with the AGP–bleomycin interactions but were too small for a quantitative estimation of the Kd, which was determined by fluorescence.

The determined values were, however, tenfold bigger than those evaluated by CD in the far-UV region.

## 3. Discussion

In Figure 2A, while in the far-UV region there is no significant changes in the HSA conformation in the presence of bleomycin, in the near-UV region (Figure 2B), visible changes were observed, which revealed that Blenoxane was indeed bound to HSA as the sum of the HSA with that of Blenoxane was not superimposable to that of the observed CD spectrum of the mixture [HSA + Blenoxane] (1:1) (Figure 2B). This was also confirmed with thermal denaturation (Figure 3A) and UV photo denaturation (Figure 3B) assays that showed significant differences in both the rate and the mode of denaturation, consistent with the Blenoxane binding interactions to HSA.

On the other hand, significant conformational (Figure 3C) and tertiary (Figure 3D) structural changes in AGP were observed in the presence of Blenoxane, which is indicative of Blenoxane binding interactions to AGP. As Blenoxane is a mixture of congeners, of which bleomycin A_2_ (A2) is the main one (55–75%), followed by bleomycin B_2_ (B2) (25–32%) [30], with A2 and A5 being reported to be more toxic than B2 and the analogue peplomycin. The different trends observed for the thermal denaturation for both HSA (Figure 3B) and AGP (Figure 3D) with Blenoxane could be due to the different bleomycin congeners, inducing different pharmacokinetics, hence interacting differently with DNA [4].

In Table 1, the higher Tm and ΔH_m_ for [AGP + A2] were consistent with an increased stability to the thermal denaturation. On the other hand, the mixture of congeners in Blenoxane had an opposite effect, destabilizing the complex and making it more easily susceptible to heating than the apoprotein itself. Overall, the thermal denaturation parameters were quite similar throughout the various experiments for HSA with Blenoxane and its congeners, and this may be due to the occurrence of a weak interaction between the HSA and Bleomycin congeners A2 and B2. On the other hand, for thermal denaturations, a single Boltzmann model fits well for both [HSA + A2] and [HSA + B2] thermal plots (Figure 4B). For AGP, the difference in Tm was about 8–9 °C, which gives an indication that [AGP + A2] complex was more thermally stable than [AGP + B2] or AGP alone (Figure 4E). The UV denaturation process was associated with a first-order kinetic process. Overall, for both AGP and HSA, the presence of bleomycins appeared to slow down the rate of denaturations upon UV illumination (Figure 4B,E).

**Figure 6:**
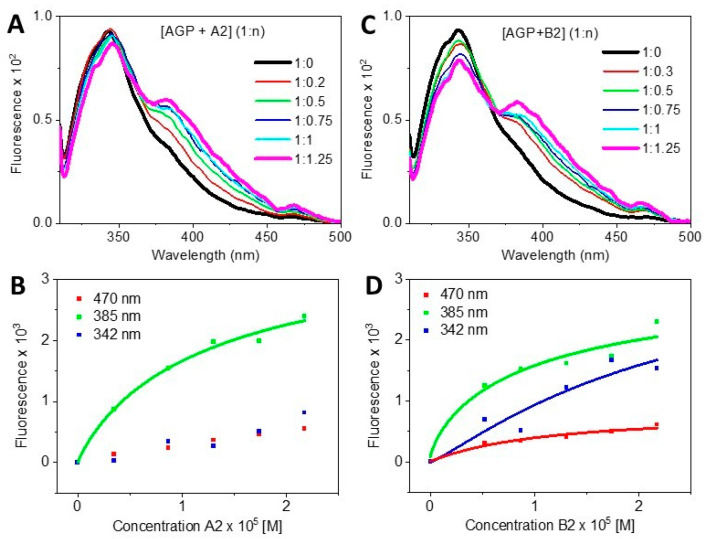
Fluorescence spectra of [AGP + A2] (1: n) titration (**A**,**B**) plots of 470 (red), 385 (green), and 342nm (blue) versus A2 concentration, and fluorescence spectra of [AGP + B2] (1: n) titration (**C**,**D**) plots of 470 (red), 385 (green), and 342nm (blue) versus B2 concentration.

**Figure 7:**
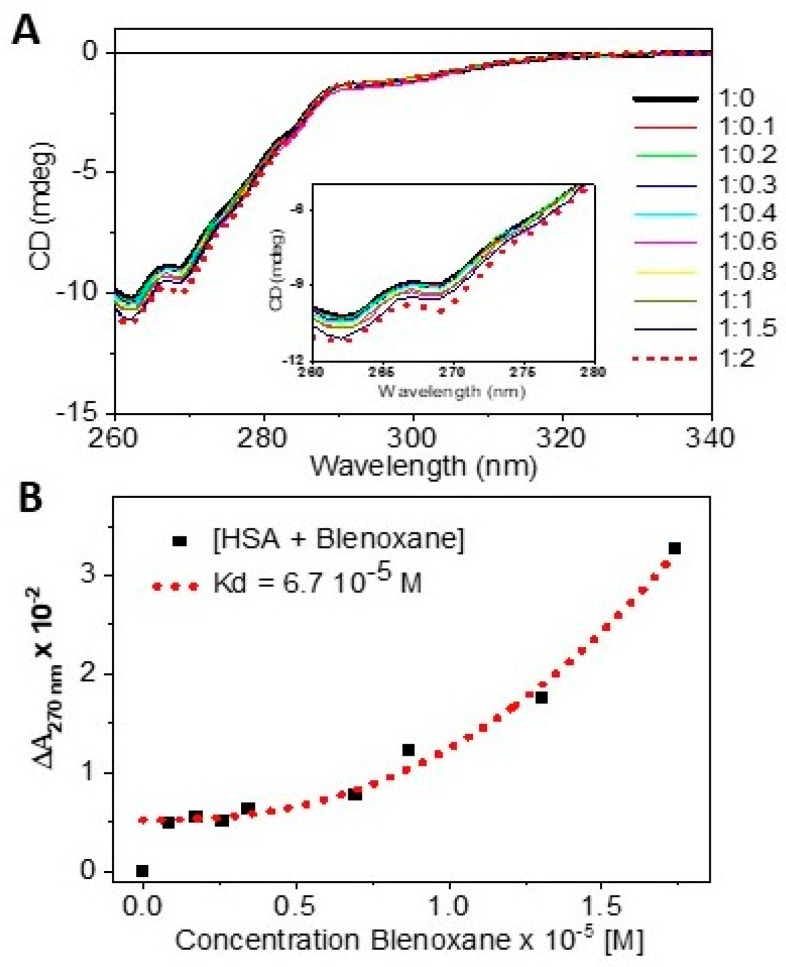
(**A**) CD titration of [HSA + Blenoxane] (1: n) molar ratio with zoomed 260–280 nm region (insert). (**B**) Plot of CD at 270 nm versus Blenoxane concentration with fit Kd of Hill equation.

This could be accounted by different interaction affinities for different parts of the molecule. With excitation at 305nm, the biggest contribution appears to be due to the bithiazole moiety, which may be less involved in the binding to AGP than another portion of the molecule. The C-terminus tails, dimethylsulphonium for A2 and guanidinium for B2, appeared to be marginally affecting the magnitude of the Kds of AGP binding, even for concentrations above 20 μM, and B2 indicated a different profile than that induced by A2. The fluorescence also displayed some differences between the two congeners that could be used for discriminating purposes (Figure 6). By plotting three wavelengths, 470, 385, and 342 nm, that correspond to the three relative maxima of the fluorescence spectra, the quasi-linear fit at 470 nm for [AGP + A2] suggested that the fluorescence of bleomycin’s bithiazole and pyrimidinyl heterocyclic rings increased proportionally. However, changes in the trends for [AGP + B2], suggesting different interactions towards AGP, might have occurred, involving the dimethylsulphonium and possibly the guanidinium. In summary, the binding of bleomycins bithiazole moiety to AGP was congener-dependent. As Bleonoxane is a mixture of congeners, care should be taken in the ratio of the congeners to preserve the activity. The fluorescence plot at 342 nm versus the ligand concentration was the most discriminating wavelength between the two AGP bound congeners (Figure 6B,D). The CD titration of Blenoxane in the near-UV region (Figure 7), unlike that in the far-UV region, was indicative of the binding interactions to HSA that were consistent with the UV and thermal denaturation results (Figure 3). A preliminary fitting of the CD data, although not reaching saturation, indicated an even larger Kd of about 67 μM (Figure 7) than those observed binding to AGP, suggesting a non-specific binding. Due to the specific and non-specific binding of bleomycin to AGP and HSA, respectively, the effective dose could be affected, with a reduced amount of free bleomycin available in vivo.

## 4. Experimental Details

### 4.1. Materials and Sample Preparations

Human serum albumin was supplied by Sigma (immunoglobulins and fatty acids free, 99.9%, A3782) (HSAff) as a lyophilized powder and used as delivered with no further purification. α_1_-acid glycoprotein (AGP) was supplied by Sigma (≥99% from human plasma, determined by agarose gel electrophoresis, G9885) as a lyophilized powder and used as delivered with no further purification.

In this study, we focused on the bleomycin congeners A2 and B2 that make up to 95% of the commercially available bleomycin (Blenoxane). Other congeners were not isolated in this work due to the low resolution provided by the purification method chosen and the very low concentration in the commercial mixture, which was produced and extracted from *Streptomyces verticillus*. Bleomycin congeners Blm-A2 and Blm-B2 were purified via silica gel flash chromatography from commercial bleomycin (Blenoxane) following a published methodology [30]. The eluted fractions were monitored with a 254 nm UV lamp on thin-layer chromatography silica plates (TLC—Merk) using Blenoxane and the reported r.f. as references. The collected fractions were dialyzed against water using Spectra-Por Cellulose Ester (CE) membranes for dialysis (MWCO 100−500 Da—Biotech) and concentrated at a low pressure and room temperature, with final concentrations of 0.160 mM and 0.180 mM (for A2 and B2, respectively). The purified fractions were characterized using ESI-MS (MW = 1414.7096 for A2, and MW = 1525.6965 for B2 at OPPF—Research Complex at Harwell, Harwell Campus, Didcot, Oxfordshire, UK). The concentrations were assessed using UV-Vis absorbance (ε^291nm^ = 17,000 M^−1^cm^−1^). For the mixture of congeners (commercial bleomycin Blenoxane), a nominal molecular weight of 1415.55 g·mol^−1^ was employed for sample preparation. The obtained solutions were stored at −20 °C in dark vials to avoid light exposure.

### 4.2. Circular Dichroism Spectroscopy

SRCD single-spectra and UV denaturation experiments were recorded on the module B unit at B23 (Diamond Light Source, Didcot, UK) using a 6-cell sample turret. The following are the experimental parameters for UV denaturation: 30 scans, 260–180 nm range, 0.5 mm slit, 1 s integration time, 1 nm increment. The thermal denaturation experiments were performed with an Applied Photophysics (APL) ChirascanPlus spectropolarimeter. All the measurements were collected in 0.02 cm quartz cells at a protein concentration of 8.7 μM for HSA and 17.4 μM for AGP with two molar ratios of Blenoxane and bleomycins A2 and B2, respectively. The baseline of water or buffer was subtracted from the CD spectra of proteins with or without ligands. Far-UV (180–250 nm) denaturation SRCD measurements were collected in 0.02 cm rotating cells with a 70 μL volume. Thermal denaturations were recorded with both B23 beamline and ChirascanPlus (APL, Leatherhead, UK). For CD titration, the protocol used was to measure the CD spectrum of each protein–ligand mixture at various ligand molar ratios by adding a small aliquot of ligand (few microliters of an appropriate concentration of ligand stock solution) to the protein in the cuvette cell. For the calculation of the Kd, the plot of the CD data of the protein–ligand mixture at the wavelength of the maximum spectral changes minus the CD of the equivalent, which used a ligand for each titration point, versus the ligand concentration were fitted using a nonlinear regression analysis of the Hill equation. The CD (Figure 2) and UV spectra of the ligand alone were used to correct the data [31]. The same method was used for the titration via fluorescence spectroscopy.

### 4.3. Fluorescence Spectroscopy

Fluorescence spectra were collected using an emission detector module of the ChirascanPlus spectropolarimeter (APL), fixing the PMU of the detector at 600 mV. For every measurement, a 1 cm quartz cell, which is suitable for CD and fluorescence, was used. Experimental details: 1 cm cell pathlength, 500–310 nm emission scan, λ_exc_ 305 nm, and 5 nm excitation bandwidth.

### 4.4. Data Analysis

The CD spectra were processed using CD Apps [27] and OriginPro. The data were reported in ellipticity (mdeg) or ΔA = (A_L_ − A_R_) at 191 nm for HSA and 195 nm for AGP and fitted with a single decay exponential for UV denaturation, using a Boltzmann curve for thermal denaturation and Hill equation for the CD titrations. The evaluated parameters of melting temperature (T_m_) were reported in °C and half-live (t_1/2_) for photo degradation in s (y = A1exp (−x/t1) + y0, t_1/2_ = t_1_log2). The thermal and photo UV denaturations and titration experiments were processed using CDApps [27]. The Hill fitting was performed with OriginLab. The secondary structure estimation (SSE) was performed with CDApps on all far-UV CD datasets. The reference dataset SDP48, SP43 + 5 Denatr (database consisting of 43 resolved soluble proteins *plus* 5 denatured proteins) was employed using the CONTIN algorithm. The elements of the secondary structure estimated from the SRCD data were labelled H1 for α-helix, H2 for distorted helix, H for overall helix, S1 for β-strand, S2 for distorted β-strand, S for overall strand, T for turns, and U for unordered.

### 4.5. Determination of Thermodynamic Parameters for Thermal Denaturation Experiments

The evaluation of molar enthalpies (ΔH_m_) and entropies (ΔS_m_) at the melting temperature for the melting process was calculated assuming a two-state native-to-unfolded model. The molar ratios at different temperatures were determined using the linear extrapolations of *plateau* at T >> T_m_ and T << T_m_ for the Boltzmann fitting curves, assuming that only denatured protein was present at T >> T_m_ and only native at T << T_m_. The reversibility was assumed to be around T_m_ ± 5 °C, and the relative molar ratios for native and denatured species was used to determine the equilibrium constants and the associated Gibbs free energy (ΔG_m_, from ΔG_m_ = −RT·lnK). Following the given relationship, ΔG_m_ was supposed to be ~0 at the T_m_ (K^(Tm)^ ~ 1). ΔH_m_ and ΔS_m_ were calculated at T_m_ by interpolating with a linear model the ±5 °C region of the ΔG_m_ vs. T curve around T_m_, assuming the intercept corresponding to ΔH_m_, previous correction from °C to Kelvin, and the slope to −ΔS_m_, assuming a *y = a − bx* model, where ΔG_m_^(Tm)^ = ΔH_m_^(Tm)^ − T_m_ΔS_m_^(Tm)^ at ΔG_m_^(Tm)^ = 0.

For the thermal denaturations, Boltzmann fitting with the equation y = y0 + A * (frac/(1 + exp ((x − x01)/k1)) + (1 − frac)/(1 + exp((x − x02)/k2))) was used. The SRCD spectra as a function of temperature were recorded with B23 module B with synchrotron operation at 300 or 350 mA, using rotating cell for thermal denaturation with 0.02 cm cell pathlength, 0.5 mm slit, 1 sec integration time, and 1 nm increment in the 260–180 nm spectral region.

## 5. Conclusions

The interactions of several bleomycins, commercial Blenoxane (the main mixture of A2 and B2 congeners) and two isolated congeners A2 and B2, with human serum albumin (HSA) and human acid glycoprotein (AGP), which are the most abundant plasma proteins responsible for small drug transport, were investigated using CD and fluorescence spectroscopies. Far-UV SRCD is an effective and fast spectroscopic tool used to determine ligand binding interactions via changes in host–ligand titrations at room temperature, thermal stability [23], and UV photo denaturation [24]. This study revealed that these bleomycins bind very differently to HSA and AGP. The CD titration in the far-UV region did not show any appreciable change in HSA folding upon the addition of bleomycins, indicating that any binding interaction, which was determined in the near-UV region, would not affect the protein secondary structure. AGP, on the contrary, showed a significant spectral change upon CD titration unambiguously, indicative of binding interactions upon the addition of Blenoxane, A2, and B2 congeners, for which the dissociation constants were determined to be very similar and in the micromolar range.

These interactions were confirmed by the thermal stabilities of HSA and AGP with and without Blenoxane, and that A2 and B2 showed different behaviors, for which the thermodynamic parameters ΔH_m_ and ΔS_m_ were consistent with the unfolding of the proteins. Both the near-UV and far-UV CD titrations revealed cooperative bindings. Monitoring the interactions in the near-UV region showed a decreased affinity of about ten times weaker compared to that monitored in the far-UV region, suggesting that AGP undergoes initial conformational changes in the secondary structure, followed by the interaction with aromatic side chain residues of AGP. Though these results are carried out in vitro, overall, these data indicate unambiguously that bleomycins bind to plasma proteins, with a stronger affinity for AGP. HSA and AGP constitute the most important plasma binding proteins; therefore, understanding their binding property with drugs is crucial in in vivo pharmacokinetic studies, especially with an increased concentration of AGP in cancer patients up to 10 times compared to healthy people [25]. The implication would be that the effective dose of free bleomycin in vivo could potentially be reduced and fall below the therapeutic margin in cancer therapy, and this ought to be considered for the optimization of the drug dosage.

## Figures and Tables

**Table 1:** Evaluated thermodynamic parameters for the thermal denaturation process at 195 nm for AGP and 191 nm for HSA. *[A]* Boltzmann fitting of experimental data. *[B]* Evaluation of T/°C, at which ΔG_m,Tm_ = 0 (T_m_) and linear fitting of the nearest points (intercept = ΔH_m,Tm_, slope = −ΔS_m,Tm_) after unit change from T/°C to T/K. Boltzmann fitting of HSA/bleomycin thermal denaturation was taken for 40–90 °C data; linear extrapolation was conducted on this limited dataset.

	*Tm* (°C) *[A]*	*Std. Dev.*	*Tm (°C) [B]*	*Std. Dev.*	*ΔH_m, Tm_* (kJ^.^mol^−1^)	*Std. Dev.*	*ΔS_m, Tm_* (J·K^−1^·mol^−1^)	*Std. Dev.*
**HSA**	74.1	1.8	67.3	3.6	85.8	5.8	252.3	9.9
**[HSA + A2] (1:2)**	71.3	0.8	69.1	2.1	95.8	3.5	279.9	5.9
**[HSA + B2] (1:2)**	65.0	0.9	62.2	4.3	91.0	7.7	271.6	13.4
**[HSA + Blenoxane]**	71.6	1.0	67.3	3.6	87.8	5.8	258.0	9.9
**AGP**	52.3	0.9	52.9	2.1	127.8	6.2	392.1	11.3
**[AGP + A2] (1:2)**	59.4	0.6	59.5	7.4	239.6	35.3	720.6	62.8
**[AGP + B2] (1:2)**	52.3	0.8	51.7	9.4	122.3	27.3	376.5	49.0
**[AGP + Blenoxane]**	50.6	0.9	49.5	4.4	91.7	10.1	284.4	18.5

**Table 2:** Half-lives estimated from exponential fittings of the UV denaturation plots (Figure 3B,E). Half-life expressed as t1/2 = t1 log2.

	*T_1/2_* (s)	*Std. Dev.*
**HSA**	1188	64
**[HSA + A2]**	1008	52
**[HSA + B2]**	1312	78
**[HSA + Blenoxane] (1:2)**	1143	44
**AGP**	720	83
**[AGP + A2] (1:2)**	1026	89
**[AGP + B2] (1:2)**	803	86
**[AGP + Blenoxane] (1:2)**	883	163

**Table 3:** Kd determined by CD and fluorescence for AGP interacting with Blenoxane, A2 and B2, respectively, and HSA with Blenoxane.

	Kd by CD		Kd by Fluorescence
	far-UV (180–260)	near-UV (260–340)	Excitation at 287 nm
[AGP + Blenoxane]	1.36 (R^2^ = 0.802)	--	--
[AGP + A2]	1.47 (R^2^ = 0.951)	--	13.23 (R^2^ 0.984)
[AGP + B2]	1.37 (R^2^ = 0.995)	--	13.68 (R^2^ 0.975)
[HSA + Blenoxane]		66.84 (R^2^ 0.929)	

## Data Availability

Data are contained within this article.

## Abbreviations

SRCD, synchrotron radiation circular dichroism; CD, circular dichroism; A2, bleomycin congener A2; B2, bleomycin congener B2; AGP, α_1_-acid glycoprotein (orosomucoid); HSA, human serum albumin; TLC, thin liquid chromatography; MS, mass spectrometry; ESI, electron spray ionization; TOF, time of flight; mdeg, millidegree (ellipticity unit); ΔA, differential absorption.
